# Icariin promotes angiogenesis in glucocorticoid‐induced osteonecrosis of femoral heads: In vitro and in vivo studies

**DOI:** 10.1111/jcmm.14589

**Published:** 2019-09-10

**Authors:** Huachen Yu, Ju’an Yue, Weiguo Wang, Pei Liu, Wei Zuo, Wanshou Guo, Qidong Zhang

**Affiliations:** ^1^ Graduate School of Peking Union Medical College Beijing China; ^2^ China‐Japan Friendship Institute of Clinical Medicine Beijing China; ^3^ Beijing Key Lab Immune‐Mediated Inflammatory Diseases Beijing China; ^4^ Department of Orthopaedic Surgery China‐Japan Friendship Hospital Beijing China; ^5^ Department of Orthopaedic Aviation General Hospital of China Medical University Beijing China; ^6^ Beijing University of Chinese Medicine Beijing China; ^7^ Peking University China‐Japan Friendship Institute of Clinical Medicine Beijing China

**Keywords:** bone microvascular endothelial cells, glucocorticoid, icariin, osteonecrosis of the femoral head

## Abstract

The injury and dysfunction of the femoral head microvascular endothelial cells are associated with the pathogenesis of glucocorticoid‐induced osteonecrosis of the femoral head (ONFH). Reports indicate that icariin (ICA) can enhance vascular roles and also inhibit endothelial cell dysfunction. However, it still remains unclear whether ICA can promote angiogenesis in glucocorticoid‐induced ONFH. In this study, we investigate this hypothesis through in vitro and in vivo experiments. Results showed that 0.1 mg/mL hydrocortisone significantly suppressed bone microvascular endothelial cells (BMECs) proliferation while ICA at 10^−5^ mol/L reversed this inhibition. ICA significantly promoted BMECs migration, tube formation, the angiogenesis‐related cytokines expression and the activation of Akt. Furthermore, ICA enhanced Bcl‐2 expression but diminished Bax expression. According to in vivo results, rats with ICA treatment exhibited a lower ratio of empty lacunae, higher volume of blood vessels and more CD31‐positive cells. This study revealed that ICA promotes angiogenesis of BMECs in vitro and improves femoral head blood vessel volume of rats treated with glucocorticoid, suggesting the efficacy of ICA in the prevention of glucocorticoid‐induced ONFH.

## 1 INTRODUCTION

Glucocorticoids are conventionally employed in the treatment of autoimmune and inflammatory diseases, such as nephrotic syndrome, systemic lupus erythematosus and rheumatoid arthritis. However, the osteonecrosis of the femoral head (ONFH) may occur following high‐dose glucocorticoid treatment.^1^, ^2^, ^3^ In China, 55.75% female ONFH patients and 26.35% male ONFH cases were found to be glucocorticoid‐induced according to a recent multi‐centre investigation.^4^ The destruction and the resultant dysfunction of hip joints requiring total hip arthroplasty may occur when ONFH is not treated properly. Even so, the majority of patients suffering from ONFH are normally young and are in constant need of multiple surgeries over their entire life‐time. Therefore, it is important to elucidate the pathophysiology of glucocorticoid‐induced ONFH and device ways of preventing its occurrence.

Although clinic glucocorticoid use and ONFH occurrence are associated strongly, the exact mechanisms are still unclear although some factors such as coagulation abnormalities, hyperlipidaemia, oxidative stress and endothelial dysfunction are suggested to be involved.^5^, ^6^, ^7^, ^8^, ^9^ These factors are thought to be associated with bone ischaemia due to the interruption of bone‐vascular supply. Moreover, reports suggest that glucocorticoids can directly harm the endothelial cells, causing in vasoconstriction, thrombus formation in the femoral head and the disturbance of the coagulation‐fibrinolysis system. Collectively, these events decrease the blood supply to the trabecular bone and finally lead to ONFH.^10^, ^11^ Therefore, maintaining the blood circulation within the femoral head is a key step to preventing glucocorticoid‐induced ONFH.

Thus, it is possible that pro‐angiogenesis substances can prevent ONFH by inhibiting endothelial cell injury. As a flavonoid isolated from Epimedii Herba, Icariin (ICA; C_33_H_40_O_15_) is a major pharmacological active component of this plant. For thousands of years, ICA has been widely used in China, Japan and Korea as aphrodisiacs, tonics and anti‐osteoporosis as recorded in the Chinese pharmacopeia.^12^ From earlier reports, ICA has been found to be an effective treatment of bone metabolic‐related diseases. Indeed, it enhances bone healing^13^ and prevents osteoporosis^14^ and ONFH.^15^ Furthermore, it was reported vascular function can be promoted by ICA as well as inhibiting the endothelial cell dysfunction.^16^ Chung et al^17^ found that ICA could stimulate in vitro endothelial cell migration proliferation, tubulogenesis, and also promote angiogenesis in situ by activating PI3K/Akt signal pathway. The protective effect of ICA on vascular endothelial cells may be associated with its anti‐apoptosis effect.^16^ However, to our knowledge, there is no study on whether ICA can prevent ONFH by improving the blood supply and promoting angiogenesis.

Given the pro‐angiogenesis and other biological functions of ICA, we have been suggested that ICA might enhance the blood supply and promote angiogenesis in ONFH. In vitro and in vivo experiments were used to test this hypothesis.

## 2 MATERIALS AND METHODS

### 2.1 Cell culture and treatment

As reported in our previous study, the bone microvascular endothelial cells (BMECs) were acquired from patients undergoing hip arthroplasty as a result of femoral neck fractures.^18^ Briefly, Dulbecco's modified Eagle's medium (DMEM; Gibco) was used to wash the aseptically collected cancellous bone (subchondral region) of the femoral head twice for 5 minutes. The supernatant was removed, and the bone debris warmly digested with 0.2% of type I collagenase (Sigma) for half an hour before they were separated with 0.25% Trypsin‐EDTA (Gibco, Invitrogen) for 5 minutes. DMEM was then added to inactivate the enzyme solution. Filtration was conducted using a 70‐μmol/L cell strainer, followed by centrifugation of the solution 430*g* for 6 minutes after which the supernatant was discarded. Cell culture was performed using endothelial cell medium (ECM; ScienCell) which contained 5 mL recombinant human vascular endothelial growth factor, 5% foetal bovine serum (FBS) and antibiotics at 37°C in 5% CO_2_. After every 3 days, the culture medium was changed. Upon reaching 80%‐90% confluence, the cells were passaged. The BMECs from two to five passages were used in all experiments. The expression of the endothelial cell markers CD31 and vWF was examined by immunofluorescence. The Institutional Ethics Review Committee of the China‐Japan Friendship Hospital approved this study. The approved guidelines and regulations were used in all experiments on BMECs, and informed consent was obtained from all study patients.

### 2.2 Cell proliferation and viability

ICA (purity > 98%) was obtained from Solarbio. Dimethyl sulfoxide was used to dissolve it, and then, the solution was stored in the dark at −20°C. The effect of ICA and hydrocortisone (HC) on BMECs proliferation and viability was evaluated using the CCK‐8 assay kit in accordance with the manufacturer's instructions. 100 μL of ECM and 10 μL of CCK‐8 solution were added to each well after treatment in 96 wells, and an additional 2 hours of incubation was performed. The micro‐plate reader at 450 nm was used to determine the absorbance value.

### 2.3 BMECs migration assay

The effect of ICA and HC on BMECs migration was evaluated after performing transwell and wound‐healing assays. For the wound‐healing assay, 5 × 10^5^ BMECs were cultured in 6‐well plates up to 24 hours until they grew to the required confluence. Thereafter, they were treated with ICA or HC for 24 hours. Using a 200‐μL pipette tip, a scratch was created on a cell monolayer. At 0 and 24 hours, the width of the scratch was measured, and the percentage of scratch recovery were determined. For the transwell assay (Corning, 8 μm), 5 × 10^5^ BMECs were suspended in 200 μL serum‐free medium in the upper chambers and then treated with different factors. About 500 μL ECM comprising of FBS was put in the lower chamber. The upper chambers were washed with phosphate buffer saline (PBS), and the cells on the top surface of this chamber were scrubbed off with a cotton swab at the end of a 12‐hour incubation at 37°C in 5% CO_2_. This was followed by fixation of the cells on the bottom surface of the membrane with paraformaldehyde (4%) for 20 minutes, followed by staining with 1% crystal violet for half an hour. The invasive cells were evaluated and counted under an optical microscope.

### 2.4 BMECs tube formation assay

After coating with 100 μL of Matrigel (BD, USA), the 96‐well plate kept under 37°C for 1 hour to allow it to solidify and polymerize. BMECs were pre‐treated with ICA or HC for 24 hours with FBS‐free condition ECM. Thereafter, seeding of a total of 2 × 10^5^ cells/well was performed on the Matrigel. Tube formation process was monitored by microscopic visualization at the end of a 6‐hour incubation and later quantified by NIH ImageJ.

### 2.5 Immunofluorescence staining

BMECs were embedded on round coverslips before placing them in a 12‐well plate. Paraformaldehyde (4%) was used to fix the BMECs for 20 minutes after reaching the desired confluence. Then, 0.1% Triton X‐100 was used to treat them for 15 minutes, and blocking was done for 30 minutes at 37°C using 10% FBS. Subsequently, using rabbit antibodies against CD31 (Abcam, 1:200) and vWF (Abcam, 1:200), the slips were labelled at 4°C overnight. On the following day, PBS was used to wash the slices three times and Alexa Fluor™488 secondary antibodies (Invitrogen) were employed to immerse them for 1 hours at 37°C. Then, 5μg/mL 4',6‐diamidino‐2‐phenylindole (DAPI) was used to stain the slips for 30 seconds, rinsing done by PBS after which a confocal laser scanning microscope was used to analyse the results.

### 2.6 Western blot analysis

After treatment of BMECs for 24 hours, proteins were extracted by RIPA lysis buffer and BCA assay kit (Beyotime) was applied for the measurement of total protein concentration. Thereafter, heating at 95°C for 5 minutes resulted in the denaturing of the proteins. SDS‐PAGE was used to separate 30 μg sample of proteins, and then, they were transferred to PVDF membranes. Blocking of the membranes with 5% dried skimmed milk was done and then incubated by primary antibodies against VEGF (Abcam, 1:1000), Akt (Abcam, 1:1000), p‐Akt (Abcam, 1:500), Bax (Abcam, 1:1000), Bcl‐2 (Abcam, 1:500) and β‐actin (Abcam, 1:3000) overnight at 4°C followed. Afterwards, incubation with their corresponding secondary antibodies was conducted. The bands were observed with Electrochemiluminescence Plus Reagent (Invitrogen). Image Lab 3.0 software was used to quantify the band intensity.

### 2.7 The qRT‐PCR assay

TRIzol reagent (Invitrogen) assisted in obtaining the total RNA extracts, and cDNA (Takara, Japan) was used to synthesize by the 1ug of total RNA. The SYBR Green system (Bio‐Rad) was used to perform qPCR. At a temperature of 95°C for 3 minutes, amplification of cDNA samples included 40 cycles of 95°C for 15 seconds and 60°C for 45 seconds. Table 1 shows the forward and reverse primers. Target gene expressions were normalized to the housekeeping gene β‐actin and calculated by the 2^−△△Ct^ method.

**Table 1:** Real‐time PCR gene markers

Gene markers	Forward	Reverse
VEGF	CCCACTGAGGAGTCCAACAT	AAATGCTTTCTCCGCTCTGA
CD31	TGTCAAGTAAGGTGGTGGAGTCT	AGGCGTGGTTGGCTCTGTT
vWF	GGGGTCATCTCTGGATTCAAG	TCTGTCCTCCTCTTAGCTGAA
PDGF‐B	CTTGGCTCGTGGAAGAAGGA	GCGTTGGTGCGGTCTATGA
β‐Actin	ACTTAGTTGCGTTACACCCTT	GTCACCTTCACCGTTCCA

### 2.8 In vivo studies

#### 2.8.1 Animal grouping and treatment

The Animal Research Committee of the China‐Japan Friendship Hospital approved all procedures that were carried out. Thirty SD rats, weighing 260 ± 20 g, were randomly assigned into the following groups: (a) the control, (b) the methylprednisolone (MP) group and (c) the MP + ICA group. Sequential drug administration for rats in the MP group and the MP + ICA group was performed to develop the ONFH model. The experimental rats were administered 10 μg/kg of lipopolysaccharide every 24 hours for 2 days. Then, the rats were intramuscularly injected with a dosage of 40 mg/kg MP (Pfizer Pharmaceutical, China) three times every 24 hours for 3 days as previously described.^19^ For the control group, a sham injection with normal saline administered was given to each rat. Gavage feeding of rats in the MP + ICA group with a daily dose of 60 mg/kg ICA for 6 weeks after the last injection of MP was carried out. The similar dosage of saline as the MP + ICA group was administered for rats from the control group and the MP group. At 6 weeks from the final injection of MP, all rats were killed. Femoral heads were collected for further assessment. In all groups, there was no rat dead before the assessment.

#### 2.8.2 Angiography and micro‐CT scanning

At 6 weeks post‐final injection of MP, angiography and micro‐CT scanning were utilized to assess the microstructure of the femoral heads. Briefly, after the administration of phenobarbital sodium for general anaesthesia, 4% paraformaldehyde and MICROFIL (MV‐112, Flow Tech, Inc) were perfused into the aorta ventralis of an open abdominal cavity as previously described.^20^ Then, the bilateral femoral heads were isolated following an overnight storage of rats at 4°C. After decalcification, micro‐CT scanning at a voxel of 9 microns was used to examine the samples and quantify the total vessel volume with CTVol software.

#### 2.8.3 Haematoxylin and eosin staining

Paraformaldehyde was used to fix the femoral head samples for 24 hours at 4°C and then decalcified with 10% ethylenediaminetetraacetic acid (EDTA, pH 7.4) for 40 days. After decalcification, the samples were dehydrated in different concentrations of ethanol, embedded in paraffin, cut into 5 μm and stained with haematoxylin‐eosin. Assessment of all specimens was carried out with a light microscope. In three randomly selected fields, the ratio of empty lacunae in the bone was calculated.

#### 2.8.4 Immunohistochemical analyses

Deparaffinization in xylene and rehydration through a graded series of alcohols was conducted for three decalcified specimens from each group. At room temperature for 10 minutes, endogenous peroxidase activity was blocked by 3% H_2_O_2_. This was followed by incubation of the sections overnight with the anti‐CD31 antibody (Abcam, 1:200) and then with secondary antibody for 30 minutes. Subsequently, the DAB peroxidase substrate was used to incubate the sections for 5 minutes. Counterstaining with haematoxylin, dehydration, clearing with xylene of all sections were done and then coverslipping. All images were captured using the same settings under the microscope (Nikon). Image‐Pro Plus software was used to analyse the images of immunohistochemical staining in terms of integrated option density (IOD) of the total area of trabecular bones and the target protein. Subsequently, calculation and counting of the mean density (IOD/area) were done.

### 2.9 Statistical analysis

All data are presented as the mean ± standard deviation (SD). A one‐way analysis of variance was used to compare the experimental groups followed by Tukey's post hoc test, and then, the count data was performed by Fisher's exact probability test. The SPSS version 21.0 (SPSS Inc) was used for statistical analysis. Values with *P* < .05 were regarded as statistically significant.

## 3 RESULTS

### 3.1 Culture and identification of BMECs

**Figure 1:**
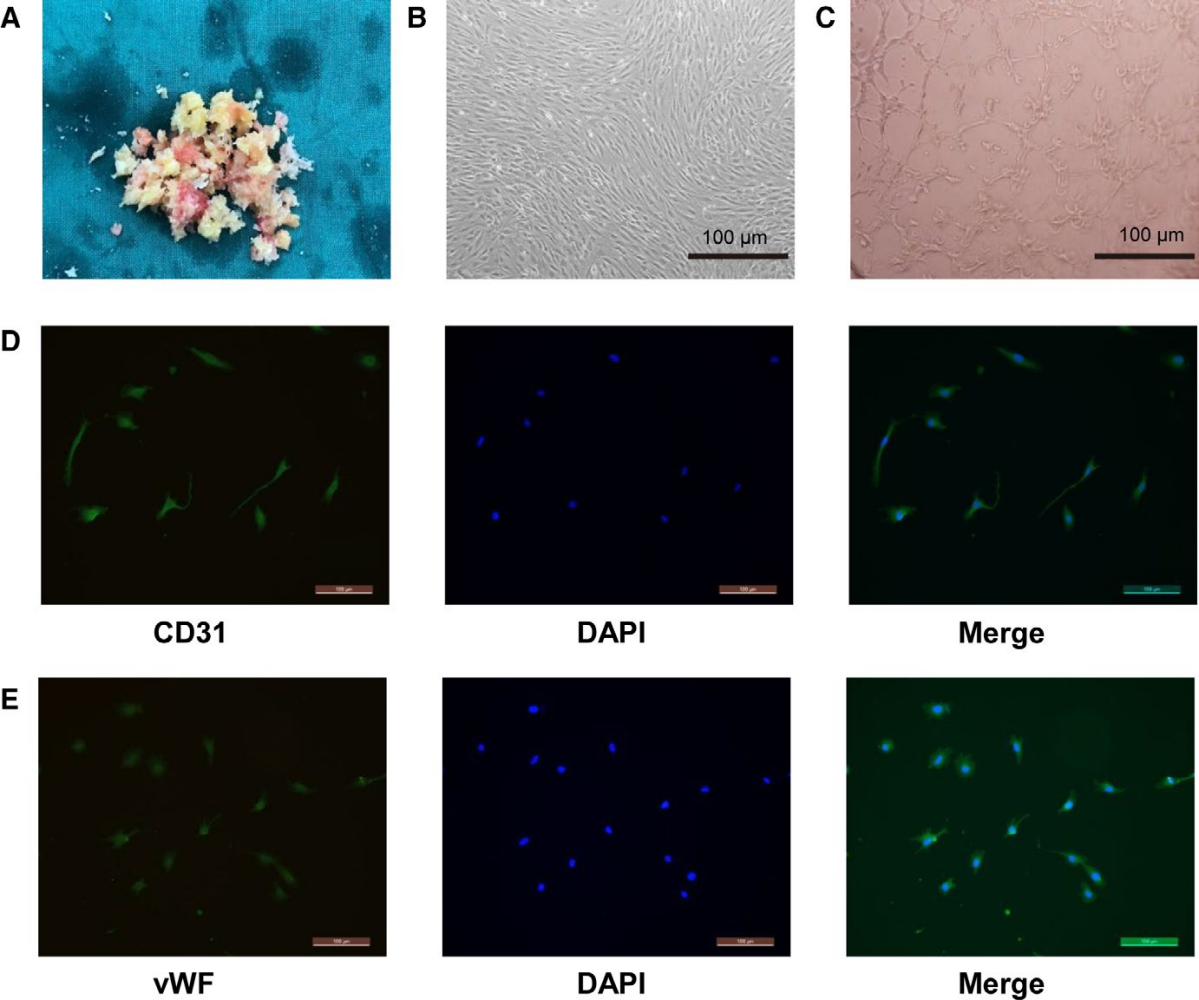
Morphology and characterization of BMECs. A, Cancellous bone in the femoral head was ground into broken bone pieces under sterile condition. B, 14‐d cultured BMECs grown to the desired confluence showing a cobblestone morphology. C, In vitro tube formation results of BMECs. Cells were grown on the Matrigel for 6 h under normal growth conditions, and capillary tube formation was observed under an inverted light microscope. (D) and (E) Immunofluorescence staining results of CD31 and vWF. The representative CD31‐positive and vWF‐positive cells were identified as BMECs

BMECs were isolated from femoral bone tissue at the subchondral region. Some cell clusters and scattered individual spindle‐shaped cells were noted 48 hours after the initial cell culture (Figure 1A,B). After 14 days of culture, the cells developed the typical cobblestone morphology of endothelial cells. The tube formation assay results confirmed the angiogenic property of the cells in vitro (Figure 1C). The isolated cells showed a high expression of CD31 and vWF (Figure 1D,E). These results indicated that these cells were BMECs and thus were used in the experiments described below.

### 3.2 Effects of HC and ICA on BMECs proliferation

**Figure 2:**
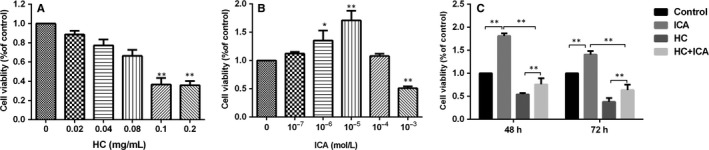
Effect of HC and ICA on BMECs proliferation. A, B, Cell proliferation results of BMECs treated with different concentrations of HC or ICA for 48 h. *indicated *P* < .05; **indicated *P* < .01. C, Cell proliferation results of BMECs at 48 and 72 h after incubation with HC (0.1 mg/mL) or ICA (10^−5^ mol/L). *indicated *P* < .05; **indicated *P* < .01

The proliferative capacity of BMECs for 48 hours by the CCK8 assay was examined by the impact of HC (0‐0.2 mg/mL) or ICA (10^−7^‐10^−3^ mol/L) on it (Figure 2A,B). HC at 0.1 mg/mL obviously suppressed cell proliferation, which is consistent with our previous studies.^21^ ICA at 10^−3^ mol/L significantly decreased cell proliferation (*P* < .01), while ICA at 10^−5^ mol/L significantly promoted the proliferation of BMECs (*P* < .01) relative to the control group. Hence, 0.1 mg/mL HC and 10^−5^ mol/L ICA were used in the following studies.

For the cell viability tests, four groups of BMSCs were established as (a) the control group; (b) the ICA group, treated with 10^−5^ mol/L ICA; (c) the HC group, treated with 0.1 mg/mL HC; and (d) the HC + ICA group, treated with 0.1 mg/mL HC and 10^−5^ mol/L ICA. As shown in Figure 2C, BMECs proliferation was significantly suppressed by HC and this inhibition was reversed by ICA.

### 3.3 Effects of HC and ICA on BMECs migration and angiogenesis

**Figure 3:**
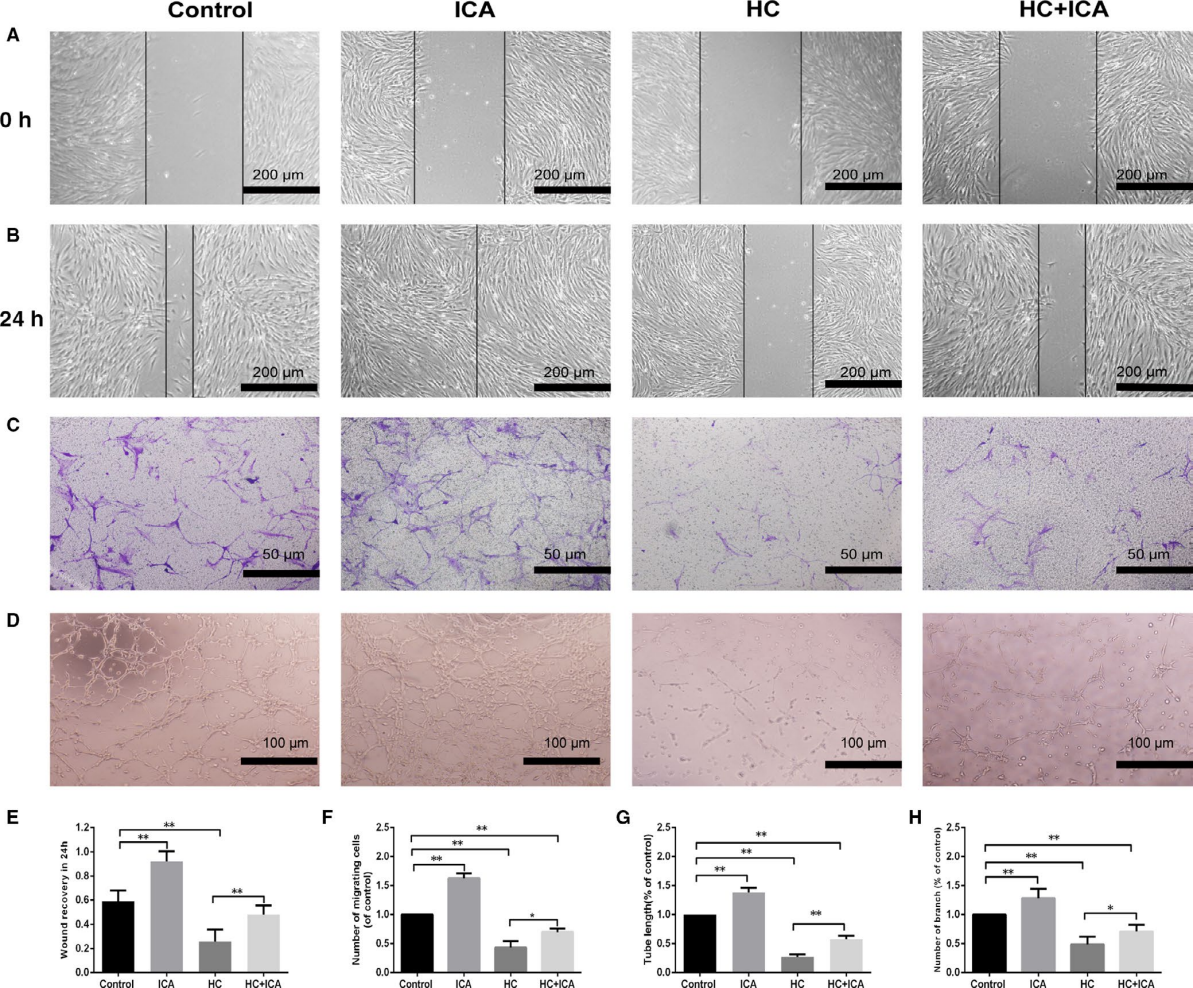
Effect of HC and ICA on BMECs migration and angiogenesis. A, B, E, Wound‐healing results of BMECs for different treatments. BMECs were treated with 10^−5^ mol/L ICA, 0.1 mg/mL HC and combination of 10^−5^ mol/L ICA and 0.1 mg/mL HC for 24 h. *indicated *P* < .05; **indicated *P* < .01. C, F, Transwell assay results of BMECs for different treatments. BMECs were treated with 10^−5^ mol/L ICA, 0.1 mg/mL HC and combination of 10^−5^ mol/L ICA and 0.1 mg/mL HC in the lower chamber for 12 h incubation. *indicated *P* < .05; **indicated *P* < .01. D, G, H, Tube formation assay results of BMECs for different treatments. BMECs were treated with 10^−5^ mol/L ICA, 0.1 mg/mL HC and combination of 10^−5^ mol/L ICA and 0.1 mg/mL HC for 6 h of incubation on the Matrigel.*indicated *P* < .05; **indicated *P* < .01

We performed in vitro tests for migration, wound healing and tube formation to determine whether ICA affects migration and angiogenesis of BMECs in the presence of HC. HC inhibited the wound recovery and migration of BMECs (both *P* < .01) as confirmed by the results of wound‐healing and migration assay, while ICA rescued the wound recovery process and migration of BMECs (*P* < .05 and *P* < .01, respectively). It was also found that BMECs treated with ICA alone showed higher migration and wound recovery compared to the control group (Figure 3A‐C,E,F).

In the tube formation assay, a smaller tube length and no loop formation were seen in the HC group. But more visible and longer tubes were observed in the HC + ICA group. Besides, we observed that BMECs treated with ICA alone had better angiogenesis activity compared to the control group (Figure 3D,G,H).

### 3.4 ICA promoted angiogenesis‐related cytokine expression as well as the activation of Akt in the BMECs

**Figure 4:**
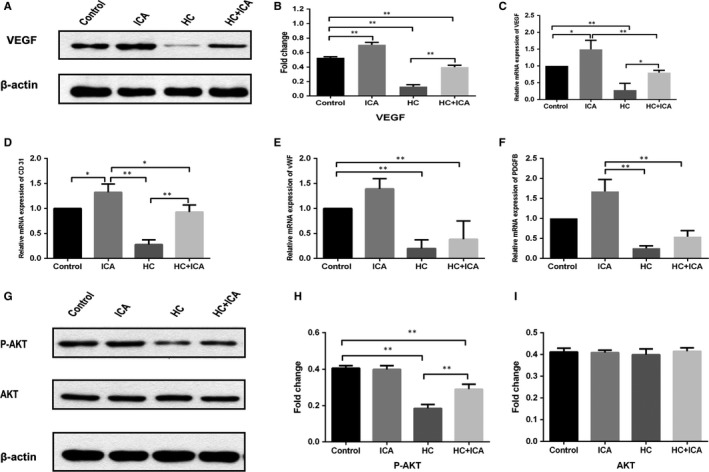
Effect of ICA on angiogenic cytokine expression and the activation of Akt in BMECs. A, Western blot analysis of VEGF in BMECs following different treatments. B, The results of the Western blot were quantified, and the protein expression of VEGF was determined. C‐F, Quantitative RT‐PCR analysis of VEGF, CD31, vWF and PDGF‐B mRNA expression in BMECs following different treatments. G, Western blot analysis of Akt and p‐Akt in BMECs following different treatments. H‐I, The results of the Western blot were quantified, and the protein expression of p‐Akt and Akt was determined. Each condition was performed in triplicate, *indicated *P* < .05; **indicated *P* < .01

Using qRT‐PCR and Western blot analyses, the VEGF expression in the BMECs was measured. The mRNA and protein expressions of VEGF in the HC group were markedly lower relative to the control group. Even so, the expression of VEGF in the HC + ICA group was obviously higher compared to the HC treatment alone, Moreover, the up‐regulation of VEGF levels in ICA group was found to be relative to the control group (Figure 4A‐C).

The expression of another angiogenesis‐related mRNA in the BMECs was also explored. CD31 has an important function in the adhesion process of endothelial cells. qRT‐PCR results demonstrated that HC suppressed the CD31 expression, while ICA significantly up‐regulated its expression when either administered alone or together with HC. vWF is a highly expressed cytokine in the endothelium, and PDGF‐B is secreted from endothelial cells. Our results show that HC and ICA did not have any effects on vWF and PDGF‐B expression (Figure 4D‐F).

The potential mechanism underlying the observed effects of ICA was explored by examining the effect of ICA on the Akt signalling pathway. As shown in Figure 4G‐I, Western blotting showed that HC significantly decreased p‐Akt level in the HC alone group compared with the control group (*P* < .01). Pre‐treatment with ICA for 24 hours significantly enhanced p‐Akt expression (*P* < .01). And the expression of p‐Akt in the ICA alone did not show any change compared with the control group (*P* > .05). Total Akt levels in each group did not change.

### 3.5 ICA suppressed the Bax and Bcl‐2‐mediated apoptosis in the BMECs

**Figure 5:**
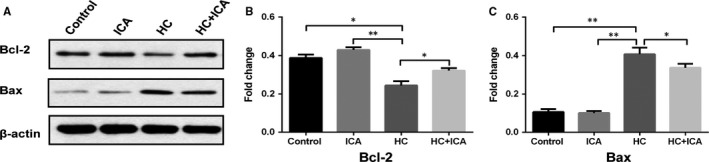
ICA suppressed the Bax and Bcl‐2‐mediated apoptosis in the BMECs. A, Western blot analyses of Bcl‐2 and Bax expression in BMECs following different treatments. B, C, The results of the Western blot were quantified, and the protein expressions of Bcl‐2 and Bax were determined. Each condition was performed in triplicate, *indicated *P* < .05; **indicated *P* < .01

The expression of Bcl‐2 and Bax in the BMECs was detected using Western blot analysis. The expression of Bax was remarkably increased (*P* < .01), while the expression of Bcl‐2 was decreased in the HC group (*P* < .05) (Figure 5) relative to the control group. However, pre‐treatment with ICA markedly reduced the expression of Bax and elevated the expression of Bcl‐2 relative to the HC group (*P* < .05). Furthermore, the protein levels of Bcl‐2 and Bax in the ICA group were comparable to those of the control group.

### 3.6 Histological analysis

**Figure 6:**
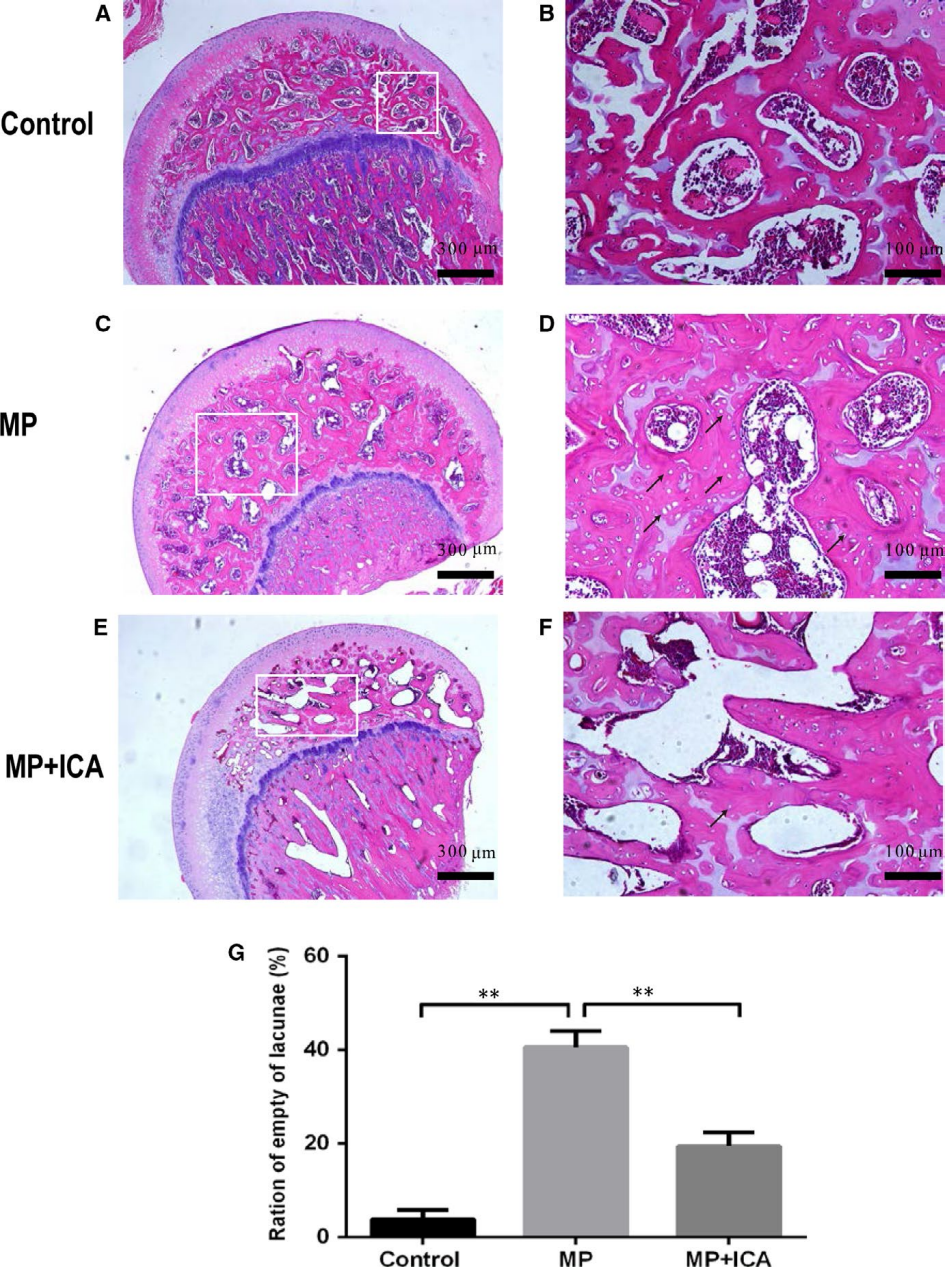
Histological analysis of rat femoral heads. No sign of ONFH was observed in the control group (A, B). Numerous empty lacunae surrounded by necrotic marrow cells were observed in the MP group (C, D), while fewer empty bone lacunae were observed in the ICA group (E, F) compared to the MP group. G, Bar shows the ratio of empty lacunae in each group. Empty lacunae are indicated by black arrows. **indicated *P* < .01

The definition of ONFH is the diffuse presence of empty lacunae or pyknotic nuclei of osteocytes in the bone trabeculae accompanied by surrounding bone marrow cell necrosis as previously reported.^22^ According to histological analysis, 2 of the 10 rats from the ICA group and 8 of the 10 rats from the MP group developed ONFH. Fisher's exact probability test showed an obvious decrease in the incidence of ONFH in the ICA groups (*P* < .05; Figure 6E‐F). No ONFH was detected in the control group (Figure 6A,B). Relative to the MP + ICA group, more empty lacunae and necrotic bone marrow cells were noted in the MP group (Figure 6C,D). The rate of empty lacunae of the ICA groups was significantly lower relative to the MP group (*P* < .1; Figure 6G).

### 3.7 ICA protected the blood vessels from the glucocorticoid‐induced ONFH in rats

**Figure 7:**
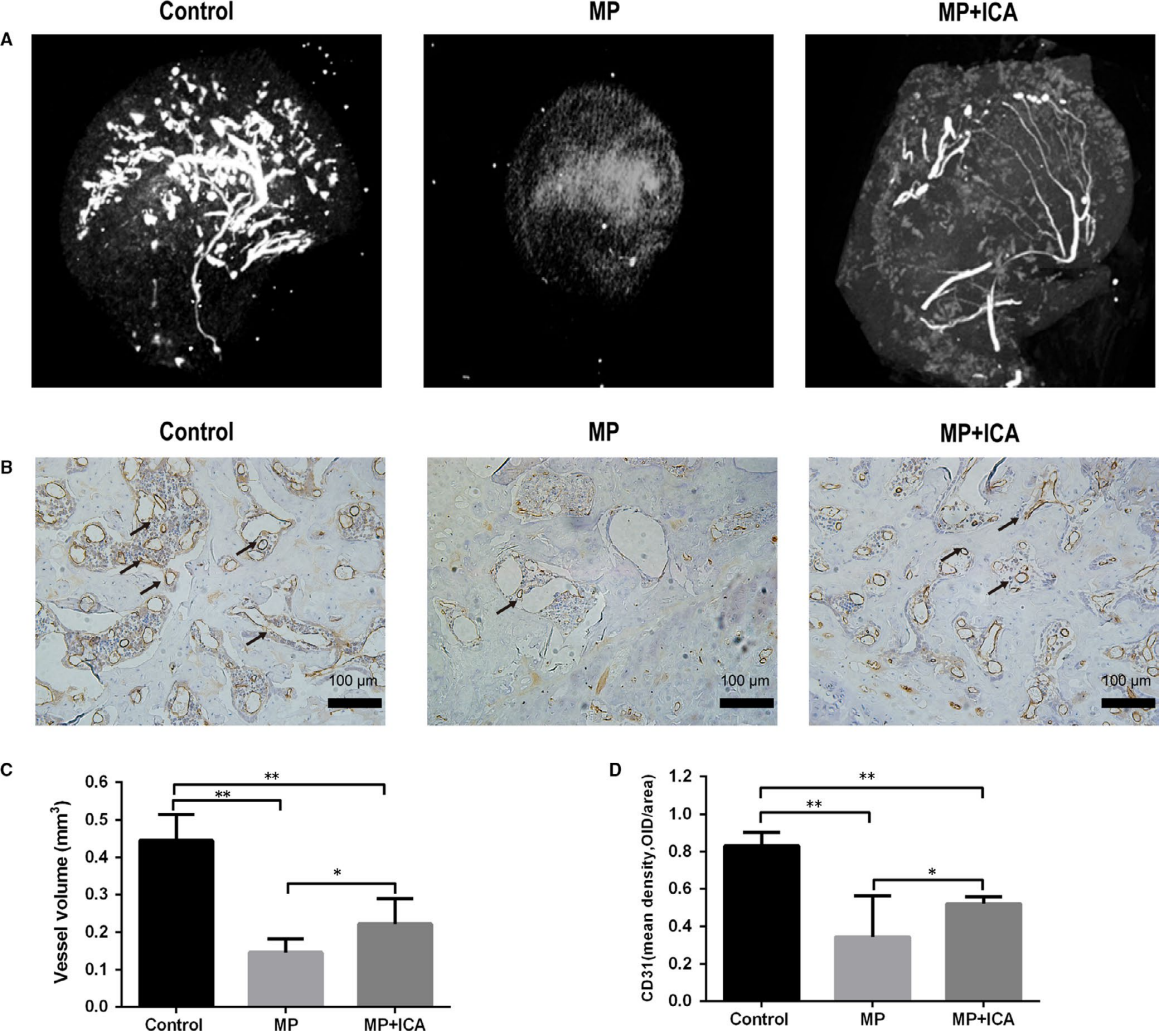
Evaluation of blood supply and revascularization in femoral heads. A, Representative images of blood vessels in the femoral heads of rats in each group, white lines represent blood vessels. B, Representative images of CD31 staining in the femoral heads of each group; the arrow indicates a CD31‐positive endothelial cell. C, Quantification of total blood vessel volume in the femoral head of each group. D, Mean density of CD31 in the femoral head slices of each group. *Indicated *P* < .05; **indicated *P* < .01

To assess the blood supply of the femoral head, we applied angiography to visualize angiogenesis in vivo. Angiographic results showed that MP markedly destroyed the blood vessels of femoral heads. However, co‐administration of ICA produced significant protective effects on blood vessels of the femoral heads (Figure 7A). Quantitatively, the MP group had significantly lower volume percentages and blood vessel volumes relative to the control and ICA groups (Figure 7C). In addition, immunohistochemical staining for CD31, a marker of endothelial cells, revealed similar results as angiography. MP significantly decreased CD31 expression, an effect that was reversed by ICA (Figure 7B,D). Therefore, ICA promotes angiogenesis in rats with ONFH.

## 4 DISCUSSION

In this study, the negative effects of HC on BMECs viability, tube formation, apoptosis, angiogenesis‐related cytokines expression and the activation of Akt through in vitro experiments were found to be reduced by ICA. We also showed that ICA administration produced significant improvement in blood vessel functions in the femoral head of glucocorticoid‐treated rats.

As studies have shown that endothelial cell injury and dysfunction are closely associated with the pathogenesis of ONFH, many studies have used human umbilical vein endothelial cells (HUVECs) to replicate the ONFH endothelial condition in vitro. Kerachian et al^23^ suggested that in addition to the heterogeneity of endothelial cells in the whole body, the endothelial dysfunction occurs at the microvascular bed in the ONFH. However, HUVECs are derived from a macrovascular bed that does not exist in adults. Therefore, in our study, we used BMECs obtained from the subchondral bone of the femoral head to enable a more specific molecular study instead of using the non‐specific HUVECs. We observed that the isolated cells formed adherent colonies and exhibited a typical cobblestone‐like morphology of endothelial cells after 14 days of culture. As previously demonstrated, endothelial cells in vitro will form capillary‐like tubules when exposed to extracellular matrix protein.^24^ Similarly, we found that BMECs formed a capillary tubular pattern when incubated on the Matrigel for 6 hours, which contains a mixture of extracellular matrix proteins. Besides, CD 31^25^ and vWF,^26^ the standard markers of endothelial cells, were highly expressed as revealed by the immunofluorescence staining. All these confirmed the presence of endothelial cells in the isolates of the subchondral bone of the femoral head.

Glucocorticoid is a widely used drug in the clinics and has been the leading cause of non‐traumatic ONFH. Seguin et al^27^ reported that ONFH and regional endothelial dysfunction are strongly associated. Glucocorticoids can directly injure endothelial cells,^10^ decrease blood flow to the femoral head^28^ and compromise microcirculation.^29^ Eventually, this leads to ischaemia and hypoxia which cause avascular necrosis of the femoral head and impairs the bone function and structure.^30^ Consistent with previous findings, our in vitro results showed that glucocorticoid inhibited angiogenesis of BMECs, including migration, wound healing and tube formation.^31^ Furthermore, we found that ICA had a protective role against the negative effects of glucocorticoid on BMECs, which demonstrates that ICA has the potential to promote angiogenesis.

VEGF has been shown to be an important cytokine in angiogenesis. It can act directly on endothelial cells to promote proliferation, migration and vascularization.^32^, ^33^ Hence, dysregulation of VEGF will have an effect on angiogenesis and consequently the repair process.^9^ In this study, we observed that HC significantly decreased VEGF expression in BMECs while ICA promoted its expression. Subsequently, we studied the expression of other factors related to angiogenesis. CD31 is mainly expressed in endothelial cells and is an endothelial marker.^25^ Endothelial cells specifically produce vWF, a marker that stains newly formed vessels.^26^ Stimulation of maturation of the vessel wall is as a result of PDGF‐B, which is secreted by endothelial cells.^34^ Compared to the HC group, our study showed that the mRNA expression level of CD31, vWF and PDGF‐B was substantially higher in the HC + ICA group. Moreover, ICA alone also increased the expression of these factors, which further supports the findings that ICA promotes angiogenesis.

The PI3K/Akt pathway has been found to regulate a wide range of fundamental cell functions such as survival, growth, migration, proliferation and cell cycle progression.^35^, ^36^ It has been shown that activation of the survival signal PI3K/Akt pathway is closely associated with vascular angiogenesis.^37^ Previous study showed that ICA stimulated angiogenesis in HUVECs by activation of Akt.^17^ Our results showed that HC decreased Akt activation and angiogenesis in BMECs, while ICA increased Akt activation and angiogenesis. These results suggest that the angiogenic activity of ICA requires the PI3K/Akt signal pathway.

Apoptosis is a normal physiological process that plays an essential role in the progression of ONFH.^38^ The endothelium is a crucial site where the control of apoptosis during angiogenesis occurs.^39^ Furthermore, the suppression of endothelial cell apoptosis is necessary for the maintenance of blood vessel integrity and for angiogenesis.^40^ Previous studies have shown that increased expression of Bcl‐2 prevents apoptosis while increased expression of Bax promotes apoptosis.^41^, ^42^ Therefore, the balance between the expression of Bcl‐2 and Bax determines cell death or survival. Feng et al^43^ found that the level of Bax protein in the ONFH group was obviously higher compared to the control group and the level of Bcl‐2 was significantly decreased in the ONFH group. This study found that ICA exerted anti‐apoptosis effects in the glucocorticoid‐induced apoptosis of BMECs by increasing the protein level of Bax and decreasing the protein level of Bcl‐2. This finding presents one of the mechanisms by which ICA promotes angiogenesis. Further studies are warranted to validate these observations.

In the in vivo study, we used LPS combined MPS techniques to develop a rat model of ONFH as these methods have been proved to be effective in modelling ONFH.^44^ The MP group showed histopathological characteristics such as empty lacunae and bone marrow cell necrosis. After the ICA treatment, the rate of empty lacunae decreased obviously. The results demonstrated that ICA can prevent glucocorticoid‐induced ONFH. In addition, we performed the micro‐CT‐based microangiography to visualize and quantify the blood circulation in the rat femoral head. We found that there were significantly more blood vessels in the ICA and control groups compared with the MP group. Furthermore, using immunohistochemical techniques, we observed that the expression of CD31, a biomarker for angiogenesis, was enhanced in the ICA group relative to the MP group, which further affirmed our hypothesis.

Our study had some limitations. First, many signal pathways regulate angiogenic activity; however, our present study only investigated the PI3K/Akt signal pathway. Further studies should investigate them. Second, the dose‐timing effects of ICA treatment on rats were not assessed. In the present study, only one dose was applied, which was based on data from preliminary studies.^45^ Whether longer treatment periods would more effective was also not conducted in this study. Further studies are needed to elucidate that.

In conclusion, this study revealed that ICA promotes angiogenesis of BMECs in vitro and improves femoral head blood vessel volume of rats treated with glucocorticoid. Therefore, it may be an effective and novel therapeutic agent for preventing glucocorticoid‐related ONFH.

## CONFLICT OF INTEREST

The authors confirm that there are no conflicts of interest.

## AUTHOR’S CONTRIBUTIONS

Huachen Yu carried out the main part of the studies and drafted the manuscript. Ju'an Yue and Weiguo Wang helped with the animal experiment. Pei Liu and Wei Zuo participated in the statistical analysis. Wanshou Guo and Qidong Zhang conceived the study, participated in its design and coordination, and helped to draft the manuscript. All authors read and approved the final manuscript.

## Data Availability

All data and materials were included in the manuscript.
